# Advances in Human Norovirus Vaccine Research

**DOI:** 10.3390/vaccines9070732

**Published:** 2021-07-02

**Authors:** Mudan Zhang, Ming Fu, Qinxue Hu

**Affiliations:** 1 Department of Gastroenterology, Guangzhou Women and Children’s Medical Center, Guangzhou Medical University, Guangzhou 510623, China; mudan@wh.iov.cn; 2 The Joint Center of Translational Precision Medicine, Guangzhou Institute of Pediatrics, Guangzhou Women and Children’s Medical Center, Guangzhou 510623, China; fuming199142@foxmail.com; 3 The Joint Center of Translational Precision Medicine, Wuhan Institute of Virology, Chinese Academy of Sciences, Wuhan 430071, China; 4 State Key Laboratory of Virology, Wuhan Institute of Virology, Center for Biosafety Mega-Science, Chinese Academy of Sciences, Wuhan 430071, China; 5 Institute for Infection and Immunity, St George’s, University of London, London SW17 0RE, UK

**Keywords:** human norovirus, acute gastroenteritis, HuNoV vaccine, VLPs, culture system

## Abstract

Human norovirus (HuNoV) is the leading cause of acute gastroenteritis (AGE) worldwide, which is highly stable and contagious, with a few virus particles being sufficient to establish infection. Although the World Health Organization in 2016 stated that it should be an absolute priority to develop a HuNoV vaccine, unfortunately, there is currently no licensed HuNoV vaccine available. The major barrier to the development of an effective HuNoV vaccine is the lack of a robust and reproducible in vitro cultivation system. To develop a HuNoV vaccine, HuNoV immunogen alone or in combination with other viral immunogens have been designed to assess whether they can simultaneously induce protective immune responses against different viruses. Additionally, monovalent and multivalent vaccines from different HuNoV genotypes, including GI and GII HuNoV virus-like particles (VLPs), have been assessed in order to induce broad protection. Although there are several HuNoV vaccine candidates based on VLPs that are being tested in clinical trials, the challenges to develop effective HuNoV vaccines remain largely unresolved. In this review, we summarize the advances of the HuNoV cultivation system and HuNoV vaccine research and discuss current challenges and future perspectives in HuNoV vaccine development.

## 1. Introduction

Human norovirus (HuNoV) is the causative agent of one-fifth of acute gastroenteritis (AGE) worldwide, with increasing frequency of outbreaks [1], which are non-enveloped, linear, single-stranded, positive-sense RNA viruses belonging to the genus Norovirus (NoV) of the family Caliciviridae [2]. According to the updated classification, NoVs are divided into 10 genogroups (GI–GX) that are subdivided into 49 genotypes [3], of which GI, GII, and GIV cause illness in humans [4]. HuNoV spreads through the fecal–oral route, which is highly contagious, with as few as 18 virus particles being able to efficiently establish infection [5,6]. Currently, most gastroenteritis outbreaks are caused by the GII.4 genotype, although the cases caused by other genotypes of GII, such as GII.2 and GII.17, are rising [7,8,9,10,11,12].

HuNoV often breaks out in semi-closed communities such as schools, hospitals, cruisers, sanatoriums, and disaster-relief agencies [13,14,15]. All age groups, especially infants, the elderly, and immunocompromised patients, are susceptible to HuNoV. Globally, HuNoV causes an estimated 699 million cases of illness and 219,000 deaths each year, resulting in >$4 billion in direct medical costs and >$60 billion in indirect medical costs [16,17,18,19]. It is estimated that globally, there is ~18% diarrhea cases associated with HuNoV infection every year [1]. Although the World Health Organization stated that the development of a HuNoV vaccine should be considered an absolute priority in 2016 [20], unfortunately, the development of an effective HuNoV vaccine has been proven to be extremely difficult. Until now, prevention of HuNoV infection largely relies on frequent hand hygiene, limiting contact with HuNoV-positive people, and disinfecting contaminated environmental surfaces.

## 2. Advances of In Vivo and In Vitro HuNoV Infection Models

The major barrier to HuNoV vaccine development is the lack of robust and reproducible in vivo and in vitro infection models. With the progressive research on the development of HuNoV in vivo and in vitro culture systems, it has been reported that HuNoV can productively infect B cells [21,22,23] and human intestinal enteroids [24,25,26] in vitro. Regarding animal models, the pigtail macaque is currently the most promising non-human primate infection model for HuNoV [27]. Nevertheless, although many trials of HuNoV infection were performed in animal models, such as adult and suckling mice, kittens, guinea pigs, or rabbits, none were successful [28,29]. To date, only the recombination activation gene and common gamma chain-deficient (Rag^−/−^γc^−/−^) BALB/c mouse was shown to support GII HuNoV replication, through the intraperitoneal but not oral route of infection [30]. Zebrafish and pluripotent stem cell-derived organoids were reported as new models for HuNoV replication [31,32]. These efforts are enabling the development of assays to determine whether antibodies induced by vaccines can abrogate HuNoV infectivity in vitro and in vivo, which would speed the progress of candidate vaccines from preclinical to clinical trials.

## 3. Advances of HuNoV Vaccine Development

HuNoV contains three open reading frames (ORFs), with ORF2 and ORF3 encoding the major (VP1) and minor (VP2) capsid proteins, respectively [33]. The expressed VP1 can self-assemble into virus-like particles (VLPs), which are virtually indistinguishable from native virus particles [34,35,36]. The VP1 capsid monomers can be structurally divided into shell (S) and protruding (P) domains. The S domain forms the structural core, while two P domains wrap around each other to form the base unit dimer [37]. Isolated P dimers still retain functional features of virus particles, including ligand-binding and some antigenic sites [38,39]. A number of HuNoV vaccines have been designed based on VLPs or P particles due to their structural and functional features [40,41,42,43,44,45,46,47,48,49,50,51]. Several HuNoV subunit vaccine candidates based on VLPs and P particles, as shown in Table 1, are in clinical and preclinical trials, under the efforts of many scientists [52,53].

### 3.1. HuNoV Vaccines in Preclinical Studies

#### 3.1.1. Vaccines Based on HuNoV VLPs

A number of HuNoV vaccines in pre-clinical trials were designed as multivalent vaccines in order to protect against not only HuNoV but also other viruses, such as rotavirus, enterovirus 71, hepatitis E virus, or astrovirus, etc.

##### Combined with RV

Rotavirus (RV) is another major cause of gastroenteritis in children, typically inducing high levels of protective antibodies after infection [62]. In order to prevent the acute gastroenteritis (AGE) caused by HuNoV and RV, combined vaccines containing HuNoV and RV immunogens were investigated. The middle capsid VP6 of RV was selected because of its high conservative feature among group A RVs and its good immunogenicity and adjuvant effect [63], whereas only HuNoV GII.4 VLPs were initially selected in the combined vaccine. However, due to the failure of the combined vaccine in inducing neutralizing antibodies against heterologous HuNoV genotypes [40], a trivalent vaccine containing HuNoV GII.4-1999 and GI.3 VLPs and the oligomeric RV VP6 was developed. In vitro studies demonstrated that RV VP6 promoted the activation and maturation of antigen-presenting cells (APCs) and facilitated the uptake of HuNoV VLPs by APCs [45]. In vivo studies indicated that the trivalent vaccine induced type-specific IgGs and neutralizing antibodies against the binding of HuNoV VLPs to histo-blood group antigen (HBGA) receptors [54]. In BALB/c mice, monovalent VLPs alone or a bivalent HuNoV vaccine based on GI.1 and GII.4-2006a VLPs were not capable of inducing the production of HuNoV-specific antibodies, whereas the monovalent or bivalent VLPs combined with RV VP6 induced significant immune responses with high levels of HuNoV-specific antibodies [44], further indicating the critical role of VP6 in the combined vaccine.

##### Combined with EV71

A combination vaccine comprised of HuNoV GII.4 and enterovirus 71 (EV71) VLPs was designed, and its immunogenicity was compared with those of monovalent GII.4- and EV71-VLPs in mice [50]. The study showed that the bivalent vaccine elicited durable antibody responses against both HuNoV GII.4 and EV71, and the antibody titers were comparable to those induced by the monovalent vaccines, indicating that there was no immune interference between the two immunogens in the combination vaccine. More significantly, mouse sera immunized with the bivalent vaccine could efficiently neutralize EV71 infection and block the binding of GII.4 VLPs to mucin [50].

##### Plant-Expressing HuNoV VLPs

Most of the HuNoV VLPs described above were produced by recombinant baculoviruses in the insect cell line sf9, while a plant expression system was also used for HuNoV vaccine development. For instance, a rapid, high-yield production of HuNoV GII.4 VLPs in leaves of Nicotiana benthamiana was developed [42]. In addition, an oral GII.4 VLP vaccine was produced in transgenic potatoes: 95% of volunteers who ingested transgenic potatoes showed significantly higher numbers of specific IgA-secreting cells, while 20% of subjects developed specific serum IgG, and 30% of subjects developed specific stool IgA [48]. Subsequently, the plant-based HuNoV VLP vaccine was produced in tobacco (Nicotiana benthamiana) by using an efficient tobacco mosaic virus (TMV)-derived transient expression system. The tobacco-derived HuNoV VLPs induced systemic and mucosal immune responses in mice [47]. Another tobacco (Nicotiana benthamiana)-produced HuNoV GII.4 VLP induced VLP-specific serum IgG for 56 days in intranasally vaccinated mice [46]. Together, these studies indicate that plant-based technology may have the potential to be a tool to produce or safely deliver vaccines.

#### 3.1.2. Vaccines Based on HuNoV P Particles

Apart from VLPs, the P particles derived from HuNoV VP1 were also explored as a novel vaccine candidate. In one study, a neonatal gnotobiotic pig was used as a model to evaluate the protective efficacies of HuNoV P particles and VLPs. Compared with VLPs, the P particles induced significantly higher numbers of activated CD4^+^ T cells in all tissues, interferon gamma-producing (IFN-γ^+^) CD8^+^ T cells in the duodenum, regulatory T cells (Tregs) in the blood, and transforming growth factor β-producing (TGF-β^+^) CD4^+^ CD25^−^ FoxP3^+^ Tregs in the spleen, indicating that P particles elicited stronger immune responses than did VLPs [43].

HuNoV combined vaccines based on P particles have also been developed in combination with immunogens derived from other viruses in order to block multiple potential viral infections. A combined vaccine of influenza virus M2e and HuNoV P particles was shown to induce protective antibodies against lethal challenge of influenza virus PR8 (H1N1). In addition, sera from immunized mice were able to block the binding of HuNoV VLPs and P particles to a HBGA [51]. Similar positive results were demonstrated when the RV VP8 was combined with HuNoV P particles [41,49]. Like VLPs, the P particles were immunogenic and showed HBGA-binding ability [39], informing its potential as a HuNoV vaccine candidate.

Due to the lack of an efficient in vitro culture system, there is currently no report on inactivated HuNoV vaccine research. We have constructed a HuNoV GII.4 infectious clone, a modified cell line to consistently produce the virus, and a human intestinal enteroid system to assess its infectivity. We also investigated the immunogenicity of inactivated HuNoVs in mice. The results showed that inactivated HuNoVs could induce a balanced Th1/Th2 response and the production of HuNoV-specific neutralizing antibodies (Unpublished data), informing the potential of inactivated HuNoV to be used for further vaccine study.

### 3.2. HuNoV Vaccines in Clinical Trails

As shown in Table 1, although a large number of HuNoV vaccines were discontinued in preclinical stages, several HuNoV vaccine candidates have been/are being tested in different phases of clinical trials.

#### 3.2.1. Monovalent Vaccines

A monovalent HuNoV GI.1 VLP vaccine adjuvanted with monophosphoryl lipid A (MPL) was tested in clinical trials, showing that it could induce HuNoV-specific serum antibodies in the majority of intranasally vaccinated recipients. Moreover, the risk of HuNoV infection and AGE development were significantly reduced in vaccinated recipients [55]. Further studies indicated that the vaccine induced immune responses in adults in a dose-dependent fashion [56]. However, considering the genetic diversity and frequent evolution of circulating HuNoV, further research was mainly focusing on vaccines combined with different HuNoV genotypes.

#### 3.2.2. Bivalent Vaccines

Considering the limitation of monovalent HuNoV vaccines, bivalent vaccines containing HuNoV GI and GII VLPs have also been explored. A dry powder formulation of bivalent HuNoV GI and GII.4 VLPs with an in-situ gelling polysaccharide was prepared and used to nasally immunize guinea pigs in order to assess the immunogenicity, potential immune interference, and safety. While the systemic and mucosal immunogenicity against each of the VLPs was increased in a dose-dependent manner, a boosting effect of the VLPs without immune interference after the second dose was also observed [64]. To study the immune responses and the mechanisms of GI.1 persistence, recombinant VLPs of five GI strains, including GI.1-1968, GI.1-2001, GI.2-1999, GI.3-1999, and GI.4-2000, were assessed. Peripheral blood mononuclear cells (PBMCs) from ten volunteers infected by GI.1-1968 were collected. Following stimulation with different VLPs, respectively, secreted IFN-γ from PBMCs was measured: 60% of the recipients responded to at least one GI VLPs, with only two volunteers responding to GI.1 VLPs. Importantly, four of five individuals responded more robustly to other GI VLPs in the cross-reactivity studies [65].

Another preclinical study showed that a bivalent vaccine containing HuNoV GI.1 and GII.4 VLPs was highly immunogenic and induced the production of homologous and heterologous HuNoV genotype-specific antibodies [66]. The immunogenicity of this bivalent vaccine adjuvanted with MPL and aluminum hydroxide was further confirmed in two clinical phase I studies [67,68], respectively. Antibodies against GI.1 and GII.4 rapidly increased and peaked at ~7 days after the first dosing, with no boost effect being observed after the second dosing. The HBGA-blocking antibody titer was significantly increased at all dose levels and in all the evaluated recipients [68]. The above studies revealed that a rapid immune response after the first dosing may be particularly important to control HuNoV outbreaks. Subsequently, B cell responses were assessed in participants for the safety and immunogenicity of the bivalent HuNoV GI.1 and GII.4 VLP vaccine [67]. The vaccine was intramuscularly vaccinated on days 0 and 28 to healthy adults aged 18–49 years in order to evaluate whether a single dose of the bivalent vaccine is potent enough to activate pre-existing B cell memory. The results indicated that a rapid activation of B cells and the mucosal homing phenotype of VLP-specific Ab-secreting cells (ASCs) were consistent with those in subjects orally primed by HuNoV. Through monitoring clinical conditions after challenge, the results showed that the signs and symptoms of HuNoV disease were less common and severe in vaccinated recipients than those in controls [69].

A clinical phase II trial subsequently evaluated the dosage of each immunogen to reach the best balance of tolerability and immunogenicity of this bivalent vaccine [59]. Enrolled subjects were randomly assigned to three groups and intramuscularly vaccinated with placebo or vaccines containing two different doses of GI.1 VLPs and GII.4 VLPs adjuvanted with MPL and Al(OH)_3_. The results showed that both candidate VLP vaccines were well-tolerated and induced robust immune responses. The formulation containing 15 µg GI.1 VLPs and 50 µg GII.4 VLPs displayed the best balance of tolerability and immunogenicity. Additionally, the safety and immunogenicity of different formulations of the bivalent HuNoV VLP vaccine candidate were assessed in healthy 18- to 64-year-olds [60], showing that all candidate HuNoV formulations were well-tolerated. Overall, the formulation of 15 μg GI.1 VLPs/50 μg GII.4 VLPs elicited the best balance of immunogenicity, with no clear benefit of MPL, indicating its potential for moving forward in clinical development.

Another clinical phase II trial of this bivalent vaccine was carried out in two age cohorts (1 to <4 years, and 6 to <12 months). In 6–12-month-old infants and children up to 4 years old, robust immune responses to the bivalent HuNoV VLP vaccine candidates were observed. After two doses of immunization with the formulation containing 50 μg GI.1/150 μg GII.4 VLPs, both age cohorts showed high antibody responses [61]. Whether such immune responses could confer protection remains unclear. A phase II study regarding immunogenicity and safety of the bivalent HuNoV VLP vaccine candidate in >60-year-olds was completed [70], showing that the elderly displayed no safety concerns and had similar immune responses to the vaccine candidate as the younger cohorts. Further clinical phase II trials regarding efficacy and long-term immunogenicity in adults are ongoing.

#### 3.2.3. Adenovirus Vector-Based HuNoV VLP Vaccine

A recombinant adenovirus vaccine expressing HuNoV GII.4 VLPs was developed by the Chinese Center for Disease Control and Prevention. Intranasal administration of recombinant adenovirus-expressed HuNoV GII.4 VLPs stimulates specific cellular, humoral, and mucosal immune responses in mice [71]. The mice primed with recombinant adenovirus and boosted with purified HuNoV GII.4 VLPs showed stronger humoral, mucosal, and interferon-γ responses than those immunized with VLPs prime-recombinant adenovirus boost or VLPs alone, suggesting that the adenovirus prime-VLPs boost vaccination is a more effective strategy to induce immune responses against HuNoV, which may be another promising direction to improve current HuNoV vaccine design [72].

Based on previous studies, a single-site, randomized, double-blind, placebo-controlled clinical trial of an oral HuNoV vaccine was launched to assess its safety and immunogenicity. The tablet vaccine was comprised of a non-replicating adenovirus-based vector expressing HuNoV GI.1 VLPs and a dsRNA adjuvant [57]. The results indicated that this oral HuNoV vaccine was well-tolerated and induced substantial immune responses, including systemic and mucosal antibodies as well as memory IgA/IgG.

Another similar vaccine using adenovirus-expressing HuNoV GII.4 VLPs was prepared, and it tested whether enhanced protection could be induced by the recombinant adenovirus vaccine. Based on the satisfactory results of the recombinant adenovirus, Vaxart Incorporation developed an oral NoV GI.1 vaccine tablet combined with an oral NoV GII.4 vaccine tablet. The phase Ib trial was designed to evaluate the safety, immunogenicity, and immune interference of this oral bivalent norovirus vaccine. As announced previously, all primary and secondary endpoints for safety and immunogenicity were met in the phase Ib study. Both the HuNoV GI.1 and GII.4 component of this vaccine induced robust mucosal immune responses in the majority of subjects without immune interference [58,73]. This oral HuNoV bivalent vaccine developed by Vaxart Incorporation may represent a promising vaccine candidate, although further clinical trials are still ongoing.

## 4. Discussion

A number of preclinical and clinical trial studies revealed that HuNoV vaccines were able to induce good immune responses [39,44,46,47,48,50,51,54,55,56,57,58,59,61,64,65,66,67,68,70,71,72,74,75,76,77,78,79]. Regarding protective immunity, one vaccine efficacy study in gnotobiotic pigs showed that adjuvanted HuNoV VLPs increased protection rates against viral shedding and diarrhea after the immunized animals were challenged with the homologous HuNoV genotype [80]. Nevertheless, the current understanding of protective immunity required to confer protection against HuNoV infection remains inconclusive. The immunity against HuNoV appears to be complex and is complicated by a number of factors. For instance, due to the lack of an efficient in vitro HuNoV infection system, many of the HuNoV vaccine trials studied the efficacy by assessing whether the vaccine-induced serum antibodies blocked the binding of HuNoV VLPs to HBGAs. This method certainly has limitations, because HuNoV VLPs with mutations within and around the receptor-binding domain, which had altered immunogenicity, still retained the ability to bind with HBGAs [81,82]. In addition, individuals with high titers of HuNoV-specific serum or fecal antibodies appeared to have a greater probability of infection by HuNoV than those with low titers of pre-existing antibodies [83,84,85,86]. These findings collectively highlighted that it is currently difficult to determine the precise role of antibodies induced by a vaccine in preventing HuNoV infection. Furthermore, some individuals were shown to be resistant to infection by a specific HuNoV genotype, despite the fact that they had functional alpha(1,2) fucosyltransferase, which is important for the expression of HBGAs, implying that memory immune responses elicited due to pre-exposure histories or other unknown factors might play a role [87].

The natural immunity against HuNoV is also poorly understood [88]. Although previous studies in humans showed that the protective immunity against a homologous HuNoV genotype lasted for 6 months to 2 years [83,89], the dose and the HuNoV genotype used in the challenge study did not necessarily mimic a typical natural exposure. In a follow-up modeling study, it was shown that the probable duration of post-exposure HuNoV immunity might range from 4 to 8 years [90]. This would be a huge challenge for developing an effective HuNoV vaccine. Another feature of HuNoV immunity is that the infection by one genogroup does not necessarily confer protection against the infection by other genogroups [52,91,92]. NoV GI, GII, and GIV cause illness in humans, which are subdivided into a number of different genotypes [3,4]. The predominant HuNoV genotype, GII.4, undergoes a process of evolution, whereby a new variant emerges every 2–4 years. Novel HuNoV GII.4 variants could alter their immunogenicity and consequently escape from the pre-existing HuNoV immunity in the human populations [93]. Together, the above studies indicated that the genetic diversity and frequent evolution of circulating HuNoVs might limit the durability of protection induced by HuNoV vaccines.

Differences of individuals in immune response to natural norovirus infection suggest that a robust immune response elicited by immunization in adults may not be equally efficacious in children. The immune response of immunocompromised patients should also be considered. Not until all these challenges are overcome it would be difficult to develop an effective HuNoV vaccine and corresponding immunization schedule to protect people from HuNoV infection. Therefore, future study is warranted to identify permissive cell lines for in vitro HuNoV culture, while establishment of challenge animal models is equally important. Together, such research systems would facilitate the development of an effective HuNoV vaccine in the future.

## Figures and Tables

**Table 1:** HuNoV vaccine candidates in preclinical and clinical stages.

Type	Vaccine Candidates	Development Stage	Institution
HuNoV VLPs	GI and GII.4 VLPs combined with RV VP6 [40,44,45,54]	Preclinical	University of Tampere Medical School, Finland
	GII.4 VLPs combined with EV71 VLPs [50]	Preclinical	Institute Pasteur of Shanghai, China
	Plant-expressed GII.4 VLPs [42,46,47,48]	Preclinical	Arizona State University, and Center for Vaccine Development, USA
	Monovalent GI.1 VLPs [55,56]	Clinical phase I	Baylor College of Medicine, and University of Maryland School of Medicine, USA
	Adenovirus vector-based monovalent GI.1 or GII.4 VLPs, or Bivalent GI.1 and GII.4 VLPs [57,58]	Clinical phase I	Vaxart Incorporation
	Bivalent GI.1 and GII.4 VLPs [59,60,61]	Clinical phase II	Baylor College of Medicine, Ghent University and University Hospital, and Takeda Incorporation
HuNoV P Particles	GII.4 P particles alone, or combined with other viruses [39,41,43,49,51]	Preclinical	Cincinnati Children’s Hospital Medical Center, Southern Medical University, and Virginia Polytechnic Institute and State University

## Data Availability

Not applicable.
